# High-dose chemotherapy followed by autologous transplantation may overcome the poor prognosis of diffuse large B-cell lymphoma patients with MYC/BCL2 co-expression

**DOI:** 10.1038/bcj.2016.99

**Published:** 2016-11-04

The concurrent expression of MYC and BCL2 on immunohistochemistry (IHC) ('double expressor') is recently emerging as one of the strongest and most unfavorable prognostic factor for diffuse large B-cell lymphoma (DLBCL).^1, 2, 3, 4, 5^ Nevertheless, the higher prevalence of older patients, together with the association between double expressor DLBCL and advanced age, may have represented a potential confounding factor affecting the conclusions of all these studies. In fact, older DLBCL patients generally have inferior outcome due to the higher risk of adverse events and difficulties to perform high-dose salvage therapies.^6^ This potential age bias was strongly suggested by a recent German study that included only young DLBCL patients at diagnosis, and did not confirm the negative prognostic effect of the double expressor phenotype after either eight cycles R-CHOEP-14 or sequential high-dose therapy followed by autologous stem cell transplant (ASCT).^7^ This discordant result may be explained by either the lower prognostic relevance of MYC/BCL2 among young DLBCL patients, and by the ability of intense chemotherapy regimens to overcome the negative prognostic value of MYC/BCL2 co-expression. In order to investigate these hypotheses, we analyzed the MYC and BCL2 co-expression clinical impact on a consecutive series of 140 DLBCL patients, 71 at diagnosis and 69 at first relapse, respectively. All patients were treated with intense chemotherapy programs oriented to final ASCT consolidation between 2003 and 2015 at the Istituto Nazionale dei Tumori Milano. Seventy-one naïve DLBCL patients (51%) were treated in first line with high-dose sequential chemotherapy integrated with monoclonal antibody Rituximab (R-HDS; Supplementary Figure S1).^8, 9^ Eligibility to this intense approach was decided considering either the high international prognostic index (IPI) score or the extended nodal and extranodal disease. The second cohort was composed by 69 (49%) DLBCL patients in first relapse after chemo-immunotherapy regimens and treated with intense salvage programs, including 53 (77%) treated with R-HDS and 16 (23%) with CORAL-like treatment (R-ICE/R-DHAP as induction chemotherapy before transplant), respectively.^10^ The patients' median age was 60 years (range, 30–76); 48 patients (34%) were older than 60 years, but all were considered fit and eligible for intense chemotherapy and transplant consolidation. The median follow-up of the whole series was 83 months (range 2–200). All pathological revisions and IHC colorations were performed as described in Supplementary Material and Methods and Supplementary Table S1.^11^ All statistical analyses were performed using appropriate scripts in R software (www.r-project.org; Supplementary Material and Methods).

Among the whole cohort of patients, 78 (55.7%) and 65 (46.4%) patients were characterized by BCL2 and MYC positivity on IHC, respectively; 38 (27.1%) were classified as double expressor DLBCL. In order to detect the real 'double hit' DLBCL patients, FISH analysis was performed only on 52/65 (80%) MYC IHC-positive patients. MYC rearrangements was detected in 8/52 (15%) patients and among them only one also harbored a BCL2 rearrangement, being classified as 'double hit' DLBCL.^1, 2, 3, 4, 5, 12^ Except for a slightly higher prevalence of patients with Eastern Cooperative Oncology Group (ECOG) score ⩾2 among non-double expressor patients, no other significant clinical differences were observed between two groups (Table 1). The overall response rate to high-dose therapy was 67% (*n*=94), and 59% (*n*=82) of all patients were transplanted according with treatment program. Sixty-eight (82%) of these were in CR at the time of transplant. Fifty-eight (41%) patients were not able to receive planned ASCT for refractory disease (*n*=47, 33%), poor mobilization (*n*=3; 2%), concurrent infections and/or toxic complications (*n*=7; 4.3%) or patient's preference (*n*=1; 0.7%). Double expressor patients did not show any higher prevalence among patients that were not transplanted considering either all patients and naive and relapsed series separately (Table 1). Myeloablative conditioning regimens were high-dose chemotherapy schedules in 63 patients (77%) and high-dose 90Y-Ibritumomab Tiuxetan in 19 (23%), respectively.^13^ Except for older age and ECOG score ⩾2, no clinical and outcome differences were observed between the two groups (Supplementary Table S2).

The overall 5-year event free survival (EFS) and overall survival (OS) were 47.2% (95% CI, 42.8–51.6%) and 63.9% (95% CI, 59.5–68.3%) respectively. In the whole cohort, double expressor patients showed a similar 5-year EFS (42.4% (95% CI, 34–50.8%) vs 49.1 (95% CI, 44–54.2%)) and OS (55.9% (95% CI, 46.7–65.1%) vs 66.4% (95% CI, 64.4–71.4%)) to other patients (Figures 1a and b). Focusing on patients older than 60 years, MYC/BCL2 co-expression did not affect EFS or OS (data not shown).

In the 71 naïve DLBCL, the 5-year EFS and OS were 65.2% (CI 95%, 59.4–71%) and 83.5% (CI 95%, 78.7–88.3%), respectively. IPI score >2 and ECOG score ⩾2 were associated with higher risk of relapse and shorter EFS (*P*<0.0001 and *P*=0.05, respectively). OS was only influenced by ECOG score ⩾2 (*P*=0.04). MYC and BCL2 aberrant expression was observed on IHC in 31 (43.6%) and 37 (52.1%) patients, respectively. Among them, 20 (28%) were characterized by BCL2 and MYC co-expression. Hans COO classification, Ki-67 expression, single MYC and BCL2 expression were not associated with inferior outcome. Conversely, the co-expression of MYC/BCL2 was associated with a trend towards worse 5-year EFS compared with the other patients in the cohort (49.4% (95% CI, 37.7–61.5%) vs 71.5% (95% CI, 65–78%); *P*=0.06) (Figure 1c). However, this higher relapse risk did not affect final 5-year OS (76.7 (95% CI, 66.4–87) vs 83.4 (78–88.8)) (Figure 1d). By multivariate analysis, only the MYC/BCL2 co-expression retained its independent prognostic power for EFS but not for OS (Supplementary Table S3).

The second study cohort included 69 DLBCL patients in first relapse after rituximab containing regimens (R-CHOP 53 (77%) and other regimens 16 (23%)). Thirty (43%) patients responded to salvage high-dose therapy and 27 (90%) were transplanted. Aberrant expression of MYC and BCL2 by IHC was observed in 34 (49%) and 41 (59%) patients, respectively. Among them, 18 (26%) were characterized by BCL2 and MYC co-expression. Overall the relapsed cohort 5-year EFS and OS were 28.8% (95% CI, 21.9–33.7%) and 39.6% (95% CI, 32.6–46.6%), respectively. Patients relapsed within 1 year from first line were associated with a significantly worse outcome in terms of 5-year EFS (15.5% (95% IC 9.9–21.1%) vs 62.2% (95% IC, 49.6–74.8%); *P*=0.0008) and OS (24.3% (95% IC 17–31.6%) vs 73.6% (95% IC, 69.6–87.6%); *P*=0.001; Supplementary Figure S2). This results confirmed what previously described by CORAL trial, and suggest that standard high-dose salvage therapies may be ineffective among early relapsed patients.^10^ The only other variable associated with inferior outcome at first relapse in terms of EFS and OS was the extranodal involvement of ⩾2 sites (*P*=0.01 and *P*=0.002, respectively). High lactate dehydrogenase (LDH) levels were associated with inferior EFS but not OS. Conversely, ECOG score ⩾2 and presence of bulky were associated with reduced survival (*P*=0.02 and *P*=0.04, respectively). All tested IHC markers did not have any clinical prognostication in terms of OS and EFS. Specifically, double expressor patients did not show any significant difference as compared with other patients in terms of 5-year EFS (37.5% (95% IC, 25.8–49.2%) vs 27.5% (95% IC, 21–34%), *P*=0.5) and 5-year OS (20.5% (95% IC, 4.6–36.4%) vs 39.4% (95% IC, 31.2–47.6%), *P*=0.8) (Figures 1e and f). In multivariate analysis, relapse in the 1 year after first-line therapy was the only variable that retained its independence for EFS and OS (Supplementary Table S4).

Overall, results of this study suggest that standard high-dose therapy and final transplant consolidation may abolish the poor prognostic value associated with MYC/BCL2 co-expression among young and/or fit DLBCL patients both in first line and first relapse. As expected the clinical outcome of transplanted and no transplanted patients was significantly different, but this was not influenced by MYC/BCL2 co-expression (Supplementary Figure S3). This suggests that MYC/BCL2 co-expression is not directly involved in refractoriness and resistance to intensive salvage therapy

Although the cutoff of ⩾40% for MYC positivity in IHC is widely accepted,^14^ different BCL2 cutoffs were used to define double expressor patients.^1, 4^ For this reason, all analyses described above were repeated using the alternative BCL2 cutoff described by Johnson *et al.* (⩾50%),^4^ and all results were confirmed (data not shown).

In our series we found a significant lower prevalence of double hit DLBCL patients compared with other series.^1, 2, 3, 4, 5, 7, 12^ This discrepancy could be due to the limited series size and to the known low prevalence of double hit DLBCL among young patients.^1, 3, 4, 12, 15^ For this reason our findings are not applicable on this distinct and aggressive biological entity.

Future incorporation of novel agents into first-line regimens may improve the efficacy and safety of intense chemotherapy regimens such as R-HDS program, therefore improving the final outcome.

## Figures and Tables

**Figure 1:**
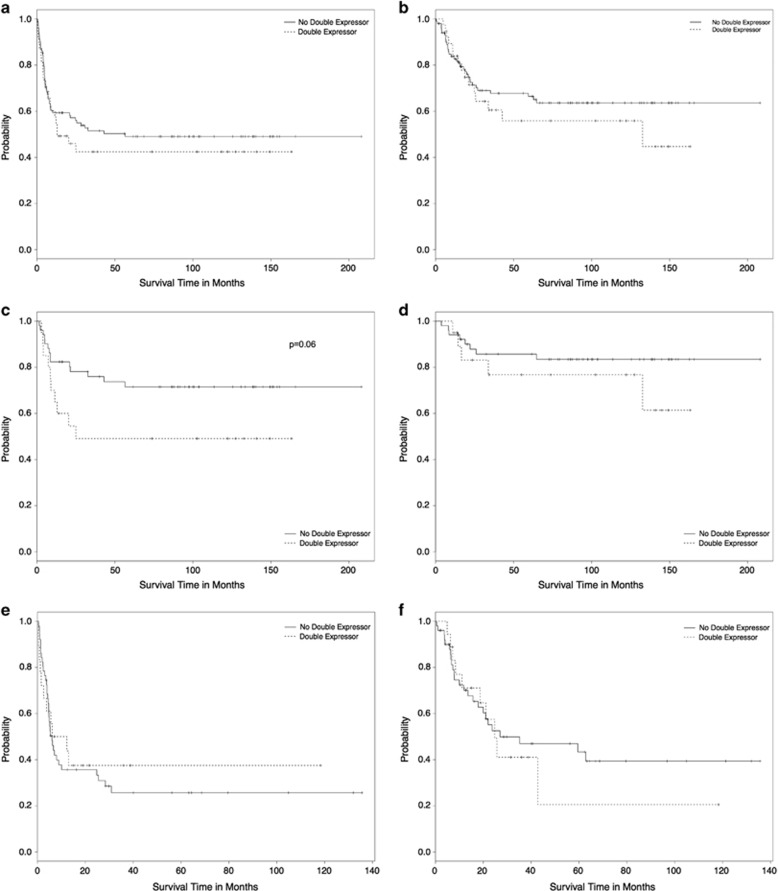
Kaplan–Meier estimated curves of EFS (**a**, **c**, **e**) and OS (**b**, **d**, **f**) according to MYC/BCL2 co-expression considering all (**a**, **b**), naïve (**c**, **d**) and relapsed/refractory (**e**, **f**) DLBCL patients.

**Table 1:** Main clinical features and treatment variables of all DLBCL treated with intense chemo-immunotherapy program with final ASCT consolidation

	*All DLBCL patients*				*Naïve DLBCL*				*Relapsed DLBCL*			
	*All*	*Double exp*	*Not double exp*	P-*value*	*All naïve pts*	*Naïve double exp*	*Naïve not double exp*	P-*value*	*All relapsed pts (69)*	*Relapsed double exp*	*Relapsed not double exp*	P-*value*
Male	84/140 (60%)	26/38 (68.4%)	58/102 (57%)	NS	39/71 (54.9%)	13/20 (65%)	26/51 (51%)	NS	45/69 (65.2%)	13/18 (72.2%)	32/51 (62.7%)	NS
Ann Arbor stage>2	110/138 (80%)	26/37 (70%)	84/101 (83%)	NS	65/71 (91.5%)	19/20 (95%)	46/51 (90%)	NS	45/67 (67.1%)	7/17 (41.1%)	38/50 (76%)	NS
Age>60	48/140 (34%)	10/38 (26%)	38/102 (37%)	NS	24/71 (33.8%)	5/20 (25%)	19/51 (37%)	NS	24/69 (34.7%)	5/18 (27.7%)	19/51 (37.2%)	0.01
ECOG⩾2	26/140 (18.5%)	3/38 (8%)	23/102 (23%)	0.05	18/71 (25.3%)	2/20 (10%)	16/51 (31%)	NS	8/69 (11.5%)	1/18 (5.55%)	7/51 (13.7%)	NS
High LDH	60/133 (45%)	13/35 (37%)	47/98 (48%)	NS	41/70 (58.5%)	12/20 (60%)	29/49 (59%)	NS	19/63 (30.1%)	1/15 (6.66%)	18/48 (37.5%)	0.01
Bulky	71/137 (52%)	21/38 (55%)	50/99 (51%)	NS	38/68 (55.8%)	12/20 (60%)	26/48 (54%)	NS	33/69 (47.8%)	9/18 (50%)	24/51 (47%)	NS
Extranodal sites⩾2	43/140 (30%)	10/38 (26%)	33/102 (32%)	NS	25/71 (35%)	6/20 (30%)	19/51 (37%)	NS	18/69 (26%)	4/18 (22.2%)	14/51 (27.4%)	NS
IPI⩾2	96/139 (70%)	22/38 (59%)	74/101 (73%)	NS	59/70 (84%)	15/20 (60%)	44/50 (88%)	NS	37/69 (53%)	7/18 (40%)	30/51 (59%)	NS
ASCT performed	82/140 (59%)	20/38 (53%)	62/102 (61%)	NS	55/71 (77%)	14/20 (70%)	41/51 (80%)	NS	27/69 (39%)	6/18 (33.3%)	21/51 (41%)	NS
												
*Conditioning*												
CT	63/82 (77%)	17/20 (85%)	46/62 (74%)	NS	39/55 (70.9%)	12/14 (85.7%)	27/41 (66%)	NS	23/27 (85%)	5/6 (83%)	18/21 (86%)	NS
HD-Zevalin	19/82 (23%)	3/20 (15%)	16/62 (25%)	NS	16/55 (29%)	2/14 (14.2%)	14/41 (34%)	NS	4/27 (15%)	1/6 (17%)	3/21 (14%)	NS
												
*Status pre ASCT*												
Complete remission	68/82 (83%)	16/20 (80%)	52/62 (84%)	NS	46/55 (84%)	11/14 (78%)	34/41 (83%)	NS	22/27 (81%)	5/6 (83%)	17/21 (81%)	NS
Partial remission	12/82 (15%)	3/20 (15%)	10/62 (16%)	NS	8/55 (15%)	2/14 (14%)	7/41 (17%)	NS	4/27 (15%)	1/6 (17%)	3/21 (14%)	NS
Stable disease	2/82 (2%)	1/20 (5%)	0	NS	1/55 (2%)	1/14 (7%)	0	NS	1/27 (4%)	0	1/21 (5%)	NS
												
*Reason for no ASCT*												
Disease	47/58 (81%)	16/18 (80%)	31/40 (77%)	NS	12/16 (87.5%)	6/6 (100%)	6/10 (60%)	NS	34/42 (81%)	10/12 (83%)	24/30 (80%)	NS
Poor mobilizer	3/58 (5.5%)	1/18 (11.1%)	2/40 (5%)	NS	0/16 (0%)	0/0 (%)	0 (0%)	NS	3/42 (7%)	1/12 (8.3%)	2/30 (6%)	NS
Adverse event	7/58 (12%)	1/18 (13.3%)	6/40 (15.5%)	NS	4/16 (25%)	0/6 (0%)	4/10 (40%)	NS	4/42 (9.5%)	1/12 (8.3%)	3/30 (10%)	NS
Other	1/58 (1.5%)	0	1/40 (2.5%)	NS	–	–	–	–	1/42 (2.4%)	0/0 (0%)	1/30 (4%)	NS
MYC>40%	65/140 (46%)	38/38 (100%)	27/102 (26%)	0.003	31/71 (43.6%)	20/20 (100%)	11/51 (21%)	<0.0001	34/69 (49.2%)	18/18 (100%)	16/51 (31.3%)	<0.0001
BCL2>70%	78/140 (55%)	38/38 (100%)	40/102 (39%)	<0.0001	37/71 (52.1%)	20/20 (100%)	17/51 (33%)	<0.0001	41/69 (59.4%)	18/18 (100%)	23/51 (45%)	<0.0001
MYC/BCL2 co-expression	38/140 (27%)	38/38 (100%)	0/102 (0%)	<0.0001	20/71 (28.1%)	20/20 (100%)	0/51 (0%)	<0.0001	18/69 (26%)	18/18 (100%)	0/51 (0%)	<0.0001
ABC-DLBCL	71/116 (61%)	22/29 (76%)	59/87 (69%)	NS	43/59 (73%)	11/14 (78%)	32/45 (71.1%)	NS	34/61 (56%)	13/18 (72%)	21/43 (49%)	NS
Ki-67 High	90/128 (70%)	27/35 (77%)	63/93 (87%)	NS	40/57 (70%)	14/17 (82%)	26/40 (65%)	NS	44/66 (66%)	11/15 (73%)	33/51 (65%)	NS

Abbreviations: ASCT, autologous stem cell transplant; LDH, lactate dehydrogenase; NS, nonsignificant.
