# Supplementary material for: High-dose chemotherapy followed by autologous transplantation may overcome the poor prognosis of diffuse large B-cell lymphoma patients with MYC/BCL2 co-expression
Source: Blood Cancer J. 2016 Nov 4;6(11):e491–. doi: 10.1038/bcj.2016.99 (PMC5148062; doi:10.1038/bcj.2016.99)
Supplement: Supplementary Information [file bcj201699x1.docx]

**Supplementary Material and Methods**

**Immunoistochemical analysis**

Two expert pathologists revised all DLBCL biopsies in order to exclude all primary mediastinal lymphoma or indolent undiagnosed forms. Formalin fixed paraffin embedding sections and colorations were performed following what was recently described.^1,2^ Cases were considered positive for c-MYC if ≥40% of tumor cells stained with antibody, whereas for BCL-2, a cut-off level of 70% positive cells was used according to what was previously described.^1^ The Ki-67 high expression was defined as >70%. The cut-off values for BCL-6, MUM-1 and CD10 were set at 30% according to the recent literature.^2^ We used CD10, BCL-6 and MUM1 staining to divide all DLBCL cases into GCB or ABC subgroups according to the Hans algorithm.^16^ Fluorescence in situ hybridization (FISH) analysis was performed using “LSI BCL2 break apart” and “LSI MYC dual color SO/SG” probes (VYSIS Abbott).

**Statistical Analysis**

The long-term outcome was assessed in terms of overall survival (OS) and event-free survival (EFS). OS was defined as the time from the start of treatment to death for any cause. EFS was defined as the time from the start of treatment to relapse. Patients that were transplanted after response to high dose therapy induction were investigated for EFS and OS after transplant, considering the date of transplant as the beginning of follow up. All contingency analyses were performed by Fisher’s exact test. OS and EFS were calculated with the Kaplan-Meier method and groups were compared with log-rank test for statistical significance. Multivariate analyses were performed with Cox models to evaluate risk factors for EFS and OS. The covariates included in multivariate analysis were based on their clinical relevance and on their prognosis role after transplant in univariate analysis. All the analyses were performed using appropriate scripts in R software ([*www.r-project.org*](http://www.r-project.org)).

**Supplementary Figure Legends**

Supplementary Figure 1 - Description of R-HDS schedule

Supplementary Figure 2 - Kaplan-Meier estimated curves of EFS (a) and OS (b) comparing patients relapsed within 1 year from first line with others

Supplementary Figure 3 - Kaplan-Meier estimated curves of EFS (a,c) and OS (b,d) according to MYC/BCL2 co-expression only patients that completed (a,b) and those that did not complete the R-HDS program
