# Supplementary material for: High-dose chemotherapy followed by autologous transplantation may overcome the poor prognosis of diffuse large B-cell lymphoma patients with MYC/BCL2 co-expression
Source: Blood Cancer J. 2016 Nov 4;6(11):e491–. doi: 10.1038/bcj.2016.99 (PMC5148062; doi:10.1038/bcj.2016.99)
Supplement: Supplementary Table 1 [file bcj201699x2.docx]

**Supplementary Table 1**. Summarization of all monoclonal antibodies characteristics used for IHC analysis

| Antibody | clone | Mono/ polyclonal | Pretreatment | Dilution | Detection System | Source |
| --- | --- | --- | --- | --- | --- | --- |
| CD10 | 56C6 | Monoclonal mouse | PTLink EDTA 15’ 96°C | 1:100 | EnVision FLEX+ Dako | DAKO |
| BCL2 | 124 | Monoclonal mouse | PTLink EDTA 15’ 96°C | 1:500 | EnVision FLEX+ Dako | DAKO |
| BCL6 | PG-B6p | Monoclonal mouse | PTLink EDTA 30’ 96°C | 1:100 | EnVision FLEX+ Dako | DAKO |
| MUM1 | MUM1p | Monoclonal mouse | PTLink EDTA 15’ 96°C | 1:50 | EnVision FLEX+ Dako | DAKO |
| c-MYC | Y69 | Monoclonal rabbit | PTLink EDTA 30’ 96°C | 1:100 | EnVision FLEX+ Dako | Abcam |
| Ki67 | MIB1 | Monoclonal mouse | PTLink EDTA 15’ 96°C | 1:400 | EnVision FLEX+ Dako | DAKO |
