# Supplementary material for: High-dose chemotherapy followed by autologous transplantation may overcome the poor prognosis of diffuse large B-cell lymphoma patients with MYC/BCL2 co-expression
Source: Blood Cancer J. 2016 Nov 4;6(11):e491–. doi: 10.1038/bcj.2016.99 (PMC5148062; doi:10.1038/bcj.2016.99)
Supplement: Supplementary Table 2 [file bcj201699x3.docx]

**Supplementary Table 2.** Clinical features of DLBCL patients transplanted with either high dose chemotherapy or high dose Zevalin

|  | Zevalin All | CT All | p value | Zevalin 2nd Line | CT 2nd Line | p value | Zevalin 1st Line | CT 1st Line | p value |
| --- | --- | --- | --- | --- | --- | --- | --- | --- | --- |
| Male | 10/19 (52.6%) | 39/63 (62%) | ns | 1/4 (25%) | 17/23 (73.9%) | ns | 9/15 (60%) | 22/40 (55%) | ns |
| Age>60 | 15/19 (79%) | 9/63 (14.3%) | <0.0001 | 3/4 (75%) | 4/23 (17.3%) | 0.04 | 12/15 (80%) | 5/40 (12.5%) | <0.0001 |
| Ann Arbor stage>2 | 16/19 (84.2%) | 53/63 (84%) | ns | 3/4 (75%) | 15/23 (65.2%) | ns | 13/15 (88.5%) | 38/40 (95%) | ns |
| ECOG>2 | 7/19 (36.8%) | 11/63 (17.4%) | 0.04 | 0/4 (0%) | 3/23 (13%) | ns | 7/15 (46.5%) | 8/40 (20%) | ns |
| High LDH | 4/19 (21%) | 31/62 (50%) | 0.03 | 1/4 (25%) | 5/22 (22.7%) | ns | 3/15 (20%) | 26/40 (65%) | 0.005 |
| Bulky | 7/19 (36.8%) | 31/63 (49.2%) | ns | 0/4 (0%) | 11/23 (47.8%) | ns | 7/15 (46.5%) | 20/40 (50%) | ns |
| Extranodal sites>2 | 2/19 (10.5%) | 12/63 (19%) | ns | 0/4 (0%) | 4/23 (17.4%) | ns | 2/15 (13%) | 8/40 (20%) | ns |
| IPI>2 | 9/19 (47.3%) | 18/63 (28.5%) | ns | 1/4 (25%) | 4/23 (17.4%) | ns | 8/15 (55.3%) | 14/40 (35%) | ns |
| MYC>40% | 7/19 (36.8%) | 24/63 (38%) | ns | 2/4 (50%) | 12/23 (52.1%) | ns | 5/15 (33.3%) | 12/40 (30%) | ns |
| BCL2>70% | 9/19 (47.3%) | 35/63 (55.5%) | ns | 3/4 (75%) | 15/23 (65.2%) | ns | 6/15 (40%) | 20/40 (50%) | ns |
| Double expressor | 3/19 (15.8%) | 17/63 (27%) | ns | 1/4 (25%) | 5/23 (21.7%) | ns | 2/15 (13.3%) | 12/40 (30%) | ns |
| ABC-DLBCL | 8/18 (44.5%) | 31/47 (66%) | ns | 2/4 (50%) | 9/19 (47.3%) | ns | 6/14 (42.8%) | 22/28 (78%) | ns |
| Ki-67 high | 12/18 (66.5%) | 34/53 (54.1%) | ns | 1/4 (25%) | 11/21 (52%) | ns | 11/14 (78%) | 23/32 (72%) | ns |
