# Supplementary material for: High-dose chemotherapy followed by autologous transplantation may overcome the poor prognosis of diffuse large B-cell lymphoma patients with MYC/BCL2 co-expression
Source: Blood Cancer J. 2016 Nov 4;6(11):e491–. doi: 10.1038/bcj.2016.99 (PMC5148062; doi:10.1038/bcj.2016.99)
Supplement: Supplementary Table 3 [file bcj201699x4.docx]

**Supplementary Table 3.** Multivariate Analysis of EFS and OS considering patients treated with R-HDS program in first line

|  | Event Free Survival | | | Overall Survival | | |
| --- | --- | --- | --- | --- | --- | --- |
|  | *P value* | *HR* | *95% of CI* | *P value* | *HR* | *95% of CI* |
| Age | 0.66 | 1.24 | 0.47-3.27 | 0.825 | 0.85 | 0.21-3.38 |
| Bulky | 0.60 | 1.28 | 0.5-3.24 | 0.90 | 0.92 | 0.25-3.36 |
| Double expressor | 0.017 | 2.95 | 1.21-7.18 | 0.063 | 3.60 | 0.96-13.77 |
| ECOG>2 | 0.36 | 1.69 | 0.54-5.25 | 0.187 | 3.08 | 0.58-16.24 |
| Extranodal sites>2 | 0.41 | 0.78 | 0.44-1.38 | 0.487 | 1.67 | 0.39-7.07 |
| IPI>2 | 0.15 | 2.48 | 0.72-8.56 | 0.366 | 2.32 | 0.37-14.38 |
| Ann Arbor Stage>2 | 0.51 | 2.02 | 0.24-16.8 | 0.998 | - | - |
