# Supplementary material for: High-dose chemotherapy followed by autologous transplantation may overcome the poor prognosis of diffuse large B-cell lymphoma patients with MYC/BCL2 co-expression
Source: Blood Cancer J. 2016 Nov 4;6(11):e491–. doi: 10.1038/bcj.2016.99 (PMC5148062; doi:10.1038/bcj.2016.99)
Supplement: Supplementary Table 4 [file bcj201699x5.docx]

**Supplementary Table 4.** Multivariate Analysis considering patients in first relapse for EFS (a) and OS (b)

|  | Event Free Survival | | | Overall Survival | | |
| --- | --- | --- | --- | --- | --- | --- |
|  | *P value* | *HR* | *95% of CI* | *P value* | *HR* | *95% of CI* |
| Age | 0.19 | 1.56 | 0.79-3.05 | 0.002 | 4.2 | 1.66-10.61 |
| Double Expressor | 0.79 | 0.89 | 0.38-2.06 | 0.87 | 0.92 | 0.34-2.46 |
| Relapse<1y | 0.003 | 4.61 | 1.69-12.62 | 0.0059 | 9.15 | 1.91-43.86 |
| Extranodal sites>2 | 0.059 | 2.06 | 0.97-4.36 | 0.32 | 2.53 | 1.08-5.91 |
| LDH | 0.02 | 2.69 | 1.18-6.16 | 0.08 | 2.45 | 0.89-6.72 |
| ECOG>2 | 0.73 | 0.84 | 0.31-2.26 | 0.04 | 2.91 | 1.03-8.19 |
| Bulky | 0.049 | 1.99 | 1.00-3.97 | 0.006 | 3.64 | 1.44-9.18 |
